# Salicylic acid as an effective elicitor for improved taxol production in endophytic fungus *Pestalotiopsis microspora*

**DOI:** 10.1371/journal.pone.0212736

**Published:** 2019-02-22

**Authors:** Kamalraj Subban, Ramesh Subramani, Vishnu Priya Madambakkam Srinivasan, Muthumary Johnpaul, Jayabaskaran Chelliah

**Affiliations:** 1 Department of Biochemistry, Indian Institute of Science, Bangalore, India; 2 Centre for Advanced Studies in Botany, University of Madras, Guindy Campus, Chennai, Tamil Nadu, India; 3 School of Biological and Chemical Sciences, Faculty of Science, Technology & Environment, The University of the South Pacific, Laucala Campus, Private Mail Bag, Suva, Republic of Fiji; Tallinn University of Technology, ESTONIA

## Abstract

Salicylic acid (SA) is an effective elicitor to increase taxol production in *Pestalotiopsis microspora*. Addition of SA at the concentration of 300 μM yielded taxol 625.47 μg L^-1^, 45- fold higher than that of the control. Elicitation of the role of SA in the fungal taxol biosynthetic pathway revealed that SA enhanced reactive oxygen species and lipid peroxidation of unsaturated fatty acids of *P*. *microspora* mycelia. This oxidative process stimulates isoprene biosynthetic pathway by triggering expression of the geranylgeranyl pyrophosphate synthase gene leading to improved biosynthesis of taxol in *P*. *microspora*.

## Introduction

Paclitaxel (taxol) is a potent anticancer drug, with a unique mechanism of action [1]. It is isolated from *Taxus* plant species and diverse endophytic fungi [2]. *Taxus* plant species are unable to meet the growing pharmaceutical demand, as the growth of *Taxus* species is relatively slow and their propagation is unsuccessful after prolonged seed dormancy. Hence, several alternative methods have been attempted to obtain taxol such as total chemical synthesis [3,4], semi-synthesis [5] and plant tissue cell culture [6]. These methods have proved inefficient due to various causes including large number of reaction steps, complex extraction methods, expensive procedures, long incubation period, low biomass, meagre yield and genetic instability. For these reasons, microbial sources of taxol production present an attractive alternative because they are simple, productive and inexpensive [1]. Endophytic fungi produce relatively lower quantities of taxol compared to plants, however yields can be improved by employing biotechnology techniques of high cell density cultivation and biomass parameter optimization and scaling up process [7]. On the other hand, biosynthesis of taxol in endophytic fungi has been rarely reported [8]. An endophytic fungus *Stemphylium sedicola* SBU-16 showed the gene of taxadiene synthase (TXS) and produced taxol and its intermediate 10-deacetylbaccatin (10-DAB) [9]. Some endophytic fungi possess taxol biosynthetic genes such as geranylgeranyl pyrophosphate synthase (GGPPS), taxadiene synthase (TXS), 10-deacetylbaccatin III-10-β-O-acetyltransferase (DBAT) and C-13 phenylpropanoid side chain-CoA acetyl transferase (BAPT), known to be present in plants [10]. The fungal taxol biosynthetic genes such as 3-hydroxyl-3-methylglutaryl-CoA (HMG CoA) reductase, taxane 5-alpha hydroxylase (T5αH), taxane 13-alpha-hydroxylase (T13αH) and taxane 2α-O-benzoyltransferase (TBT) were recognized in *Cladosporium cladosporioides* MD2 by transcriptome analysis [11]. The genes encoding GGPP synthase is of particular interest because it codes for the branch point prenyltransferase, which is involved in the formation of diterpenoid moiety, a precursor for taxol biosynthesis [12].

Elicitors can increase production of secondary metabolites by influencing the biosynthetic pathway of secondary metabolism [13]. The enhancement of taxol production strongly dependent on reactive oxygen species (ROS) [14] by elicitor-induced-lipid peroxidase, peroxidase and glucose-6-phosphate dehydrogenase (G6PDH) in *Taxus* plant cell suspension culture [15]. In addition, a study has proven the activity of taxol biosynthetic pathway key enzyme taxadiene synthase was greatly increased after elicitation in *Taxus* plant cell suspension culture [16]. Qiao et al. [17] speculated that, addition of elicitors in the fermentation medium can enhance the activity of oxidase and promote the synthesis reaction of taxol, such as cytochrome P450 oxygenases. Taxol production was increased in *P*. *microspora* when phosphate was decreased and sodium benzoate was added in the fermentation medium [18]. Supplementation of sterol biosynthesis inhibitors such as tebuconazole and triadimefon in the culture medium showed increased yield of fungal taxol [19]. Amini et al. [20] reported that squalestatin induced accumulation of H_2_O_2_ and endogenous methyl jasmonate (MeJ) but suppressed squalene synthase (SQase) activity. These were correlated with the overexpression of key genes, resulting in remarkable increase in taxane production. It may effectively inhibit SQase in competitive pathways of isoprenoids and sterols, leading to utilization of intermediate substrates for the accumulation of taxanes in yew cell culture.

Biosynthesis of salicylic acid (SA) helps the plants to establish the systemic acquired resistance (SAR) against various phytopathogens [21–23]. Exogenous SA can induce antioxidant enzyme activities, formation of pathogenesis-related (PR) proteins and expression of antioxidant enzyme genes in some plant leaves [24,25]. Mutation in PR proteins leads to decreased SA production thereby increasing the susceptibility to non-pathogenic and pathogenic microorganisms [26]. A number of plant associated endophytes, pathogenic and mutualistic fungi have the ability to suppress host defense mechanisms [27], possibly through the production of acetylsalicylate deacetylase or hydroxylase or isochorismatase, which degrade the SA into catechol. Fungus *Ustilago maydis* secretes a cytosolic acetylsalicylate deacetylase which can convert SA into catechol during the infection [28]. Similarly, the fungal phytopathogen, *Sclerotinia sclerotiorum* was reported to produce endogenous SA hydroxylase to degrade SA into catechol [29]. The plant pathogenic fungus *Fusarium graminearum* also produces SA-degrading salicylate hydroxylase [30]. However, filamentous fungi such as *Phytophthora sojae* and *Verticillium dahlia* produce isochorismatase for degradation of SA [31]. Knowledge is still limited on the direct effect of exogenous SA, on fungal growth and production of secondary metabolites [25, 32–34]. The endophytic fungus *Paraconiothyrium variabile* is reported to produce taxol at an improved yield (14.7 μg L^-1^) on supplementation of SA (50 mg L^-1^) [35] although, the mechanism of action of SA is unknown. The objective of the present study was to evaluate the use of SA as an elicitor for the improved production of taxol by the endophytic fungus *Pestalotiopsis microspora*. We also investigated the mode of action of SA on the biosynthetic pathway of taxol. To the best of our knowledge, this is the first report on role of SA in the biosynthetic pathway of taxol production in fungi.

## Materials and methods

### Strains and chemicals

*P*. *microspora* was isolated from the bark of *Taxodium mucronatum* collected from the forest at Botanical Garden (2623 m above sea level) in Tamil Nadu National Park, Ooty, India (S 10° 38ʹ 11.49ʺ; E 76° 0ʹ 77.15ʺ). The bark of *T*. *mucronatum* was cut into pieces (~0.5 × 0.5 × 0.5 cm) and surface sterilized with 70% (*v*/v) ethanol and 4% sodium hypochlorite for 60 sec to avoid epiphytic microorganisms. Small pieces of inner bark were placed on the surface of PDA medium supplemented with 150 mg L^-1^ chloramphenicol in Petri plates and incubated at 26 ± 1°C in 12 h light/dark chamber for 7 days and the Petri plates were checked regularly for the growth of endophytic fungal colonies. Pure cultures were obtained and maintained for further use. Taxol producing endophytic fungus was screened and identified as *P*. *microspora* by classical and molecular taxonomy [36]. The 28S rDNA sequence data of the *P*. *microspora* was deposited in the GeneBank database (Maryland, USA) with an accession number of HM802304. SA and the other main chemicals used in the experiments were obtained from Sigma Co. (Sigma-Bangalore, India).

### Effect of SA on the yield of taxol by *P*. *microspora*

#### Medium and culture conditions

The eight mycelia agar discs (9 mm) of *P*. *microspora* were inoculated into the M1D medium [sucrose 30 g, ammonium tartrate 5 g, yeast extract 0.5 g, soytone 1 g, Ca (NO_3_)_2_ 280 mg, KNO_3_ 80 mg, KCl 60 mg, MgSO_4_ 360 mg, NaH_2_PO_4_ 20 mg, H_3_BO_3_ 1.4 mg, MnSO_4_ 5 mg, ZnSO_4_ 2.5 mg, KI 0.7 mg, distilled water 1000 ml and pH 6.8 ± 0.2]. Each 200 mL of M1D modified medium in a 500 mL Erlenmeyer flask was cultured for 3, 6, 9, 12, 15, 18, 21, 24, 27 and 30 days incubation period at 25°C in static condition under 12 h of light and 12 h of dark cycles.

#### Biomass

After the incubation period, the fungal biomass of each flask was filtered, the mycelium was washed thrice with 0.9% NaCl (w/v) and the fungal mat was dried at 60°C for 24 h. The mycelial biomass was measured gravimetrically and reported in grams of dry weight per litre of M1D culture medium.

#### SA supplementation

Agar discs (9 mm) containing mycelia of *P*. *microspora* were inoculated in Erlenmeyer flasks (500 mL) each containing 200 mL M1D medium and incubated at 25°C in static condition under 12 h of light and 12 h of dark cycles. At the 6^th^ day of incubation, different concentrations of SA (0, 30, 60, 75, 120, 150, 240, 300, 600, 1200 and 2400 μM) were aseptically added in the respective conical flasks and continued with the incubation at 25°C under static condition in a light chamber with 12 h of light and 12 h of dark cycles. M1D medium without SA served as a control. All the experiments were carried out in triplicates with two sets. One set of cultured mycelia was harvested at the 12^th^ day to analyse genetic, biochemical and physiological changes whereas another set was harvested at the 21^st^ day to analyse taxol production.

#### Quantification of taxol

At the 21^st^ day of incubation, each 200 mL M1D medium containing *P*. *microspora* supplemented with different concentrations (0–2400 μM) of SA was filtered through double layered cheese cloth. The culture filtrates were extracted with dichloromethane (DCM) as previously described by Strobel et al. [37] and the fungal taxol was quantified as described by Gangadevi and Muthumary [38]. Briefly, each sample was dissolved in HPLC grade methanol, filtered through 0.2 μm PVDF filter and 20 μL was injected into the HPLC (Agilent compact 1120 liquid chromatography) on a C18 column monitored with UV detector at 232 nm and acetonitrile/water (25:35:40; v/v) was used as the mobile phase with a flow rate of 1 mL min^-1^.

### Electrospray ionization-mass spectroscopy (ESI-MS) analysis of fungal taxol

ESI-MS was performed for the sample from the 300 μM SA-treated fungal culture filtrate. Thermo Scientific MS system (XL/LTQ Orbitrap Discovery) coupled with a Thermo Scientific HPLC system (Accela PDA detector/autosampler/pump) was used. The following conditions were maintained in LC-MS analysis: capillary voltage 45 V, capillary temperature 260°C, auxiliary gas flow rate 10–20 arbitrary units, sheath gas flow rate 40–50 arbitrary units, spray voltage 4–5 kV and a mass range of 100–2000 amu (maximum resolution 30,000). Gradient separation was used on a Waters SunFire C18 RP analytical HPLC column (100Å, 5 μm, 4.6 mm × 150 mm) with a mobile phase of 0–100% MeOH over 30 min at a flow rate of 1 mL min^-1^.

### Estimation of oxidative and non-oxidative antioxidant enzymes

#### Preparation of mycelial homogenate

The SA-treated mycelial mat was harvested on the 12^th^ day of incubation, washed thoroughly with sterile water, cut into small pieces (approx. 1 cm), and dried using blotting paper. Two grams of fresh mycelia was homogenized at 4°C with 10 mL of 0.1 M sodium phosphate buffer (pH 7.0) using clean pre-chilled pestle and mortar. This crude homogenate was centrifuged at 10,000 rpm for 20 min at 4°C. The cell-free supernatant was sterilized using a filter membrane (0.2 μm) and stored at -20°C for further use.

#### Determination of protein

The proteins were determined by colorimetric assay according to Bradford [39].

#### Catalase (CAT)

The CAT activity was tested as previously described by Aebi [40]. Briefly, 0.5 mL of the sample was mixed with 1.5 mL of phosphate buffer (0.5 M, pH 7) and 0.5 mL of H_2_O_2_ (20 mM). The optical density (OD) was recorded using a spectrophotometer (Hitachi U-2900, Japan) at 240 nm. One unit of activity is defined as the amount of enzyme that catalyzes the decomposition of 1 μmol of H_2_O_2_ per min. Specific activity is defined as U mg^-1^ of protein.

#### Lipid peroxides (LPO)

LPO content in the mycelial mat was determined by thiobarbituric acid (TBA) reactive substance. To 0.5 mL of mycelial homogenate, 1.5 mL of 20% acetic acid, 0.2 mL of sodium dodecyl sulfate (SDS) and 1.5 mL of TBA were added and thoroughly mixed. The mixture was made up to 4 mL using distilled water and then heated at 95°C for 60 min. After cooling, 4 mL of butanol-pyridine mixture (15:1, v/v) was added and mixed well. The suspension was centrifuged at 4000 rpm for 10 min; the organic solvent layer was removed and the absorbance measured at 532 nm. TMP (1, 1, 3, 3-tetramethoxypropane) was used as a standard. The lipid peroxide content was expressed as absorbance at 532 nm in MDA mg^-1^ protein [41].

#### Peroxidase (PX)

The PX activity was performed as previously described by Claiborne and Fridovich [42]. The reaction mixture contains 0.1 M acetate-Na buffer (pH 5.5) with 4 μM of o-dianisidine in methanol and 10 mM of H_2_O_2_ as the substrate and co-substrate, respectively. One unit of PX activity was defined as the amount of enzyme which converted 1 μmol of o-dianisidine per min (ε_460_ = 11,300 M^-1^ cm^-1^) [43].

#### Superoxide dismutase (SOD)

The SOD activity was determined by monitoring the intracellular chemical reduction of nitrobluetetrazolium (NBT) and reading absorbance at 560 nm [44]. The mycelia were homogenized in 1 mL cold 100 mM phosphate buffer (pH 7.8) containing 0.1 mM ethylenediaminetetraacetic acid (EDTA), 1% (w/v) polyvinyl-pyrrolidone (PVP) and 0.5% (v/v) Triton X-100.

The reaction mixture contained, 1.9 ml of phosphate buffer (pH 7.8), 1.5 mM NBT and 0.12 mM riboflavin, with suitably diluted mycelial homogenate in a total volume of 3 ml. Illumination of the solution was carried out in an aluminium foil lined box for 10 min using 15W fluorescent lamp. Control without the enzyme source was maintained. One unit of SOD activity was defined as the amount of enzyme required to cause 50% inhibition of the reduction of NBT as monitored at 560 nm. Determination of SOD activity is expressed as U min^-1^ mg^-1^.

#### Glutathione (GSH)

The non-enzymatic antioxidant potential of total reduced GSH was determined using 5, 5’-dithiobis-2-nitrobenzoic acid (DTNB) assay. The mycelial homogenate (0.1 mL) was precipitated with 5% trichloroacetic acid (TCA) and centrifuged at 12,000 rpm for 15 min. The supernatant was mixed with 2 mL of DTNB reagent and the final volume was made up to 3 mL using phosphate buffer (0.2 M, pH 8.0). The absorbance was read at 412 nm against the control containing TCA. An aliquot of the standard solution was treated similarly. The amount of GSH in mycelia was expressed in μmole g^-1^ [45].

### Total lipid estimation and gas chromatography-mass spectrometry (GC-MS) analysis

Fresh mycelia were ground with 10 mL of chloroform: methanol (2:1, v/v) using a pre-chilled mortar and pestle. The homogenate was filtered and the filtrate transferred to a separating funnel and shaken well with 1/5 volume of 0.9% aqueous sodium chloride and kept undisturbed for 6–12 h. The bottom layer was collected. One mL of chloroform extract containing lipids was mixed with 0.5 mL of concentrated H_2_SO_4_ and placed in the boiling water bath for 10 min. After cooling, 5 mL of phospho-vanillin reagent was added, thoroughly mixed and incubated for 30 min at room temperature. Absorbance was read at 520 nm and total lipid content was expressed as mg g^-1^ of fresh weight of mycelia [46].

The control and SA treated mycelia of *P*. *microspora* were ground using a pre-chilled mortar and pestle. Lipid was extracted from the ground mycelia using chloroform and the solvent was evaporated using a rotary evaporator [47]. The dried lipid sample was re-dissolved using hexane and was subjected to GC-MS analysis (Shimadzu instrument GC: Aligent 7890 A, MS: MS detector 5975C, Ionization for MS: Electron Impact Ionization, Mass Analyzer: Single Quadrupole).

### Estimation of sterol

One gram of control (untreated with SA) mycelia and SA treated mycelia were ground with 10 mL of chloroform: methanol (2:1, v/v) using a pre-chilled mortar and pestle. The homogenate was filtered and transferred to a separating flask and shaken well with 1/5 volume of 0.9% aqueous sodium chloride. Then the separating flask was kept undisturbed for 6–12 h. The bottom layer was collected. One mL of the extracted sample was concentrated by evaporation and dissolved in 6 mL of glacial acetic acid. Then, 4 mL of ferric chloride reagent was added immediately. The contents were thoroughly mixed, cooled and the colour developed was read at 550 nm against the blank reagent. The sterol content was estimated using ergosterol as the standard (10–25 μg mL^-1^) and expressed as μg g^-1^of fresh weight of mycelia [48].

### Microscopic observation of lipid accumulation

Mycelia treated with SA (0, 150 and 300 μM concentrations) were observed for intracellular lipids using 40X in the fluorescence microscope (Zeiss AX10 Imager A2, Zeiss, Germany). The microscopic samples were prepared by mixing 1 mL of Nile blue solution (1 mg mL^-1^ methanol) with 100 μl of the culture broth. The blue light was used for excitation.

### Protein profile analysis by SDS-polyacrylamide gel electrophoresis (SDS-PAGE)

The intracellular protein concentration was determined using the method described by Bradford [39]. Briefly, aliquots of mycelial supernatants of 150, 250, 300, and 1200 μM of SA supplemented M1D media and the control (without SA supplementation) were mixed with equal volumes of loading buffer (5 mM Tris, 2.5% 2-mercaptoethanol, 1.5% SDS, 0.025% bromophenol blue and 15% glycerol) and heated in a boiling water bath for 10 min. SDS-PAGE protein profiles were obtained after electrophoresis of 10 μL of the denatured protein solutions in polyacrylamide gel with SDS in a discontinuous buffer system with 4.5% stacking gel and 12.5% running gel. The electrophoresis was performed at 125 v in a cold chamber and the protein bands present in gels were fixed in a solution of 12.5% sulfosalicylic acid for 20 min and stained with 0.025% coomassie brilliant blue G-250 for 12 h. The gels were de-stained by successive washings in acetic acid: methanol: water (1:2.5:6.5) solution.

### GGPPS gene analysis by semi-quantitative reverse transcription polymerase chain reaction (RT-PCR)

Total RNA was extracted from SA treated mycelia of *P*. *microspora* using the trizol method [49]. Isolated RNA was used as a template for first strand cDNA synthesis in a 20 μl reaction with M–MuLVRT reverse transcriptase (RT) and oligo (dT)18 primer 5ʹ-d (TTTTTTTTTTTTTTTTT)-3ʹ as the primer according to the manufacturer’s protocol. The cDNA was amplified by PCR using fungal gene specific primers of GGPPS: forward 5ʹ-AAGGCAATGGAGAAGATGTCTGG-3ʹ and reverse 5ʹ-GCTTGAGGATGTTGATGAGCTGGAG-3ʹ primers, designed (NCBI accession no. X96943) and screened using cDNA as a template. The house-keeping gene (β-actin gene) using the specific primers act-F (5'-GTGACAATGGAACTGGAATGG-3') and act-R (5'-AGACGGAGGATAGCGTGAGG- 3') were designed according to the conserved regions of mycelium actin genes. The PCR reaction mixture was prepared on ice: 10X PCR buffer, plus 1X Mg^2+^ (10 mM Tris-HCl, pH 8.5, 50 mM KCl, 1.5 mM MgCl_2_), 10 mM dNTP mixture to final concentration of 0.2 mM each, primer mix to final concentration of 0.2 μM each, template cDNA to final concentration of 100 ng and Taq DNA polymerase to final concentration of 2.5 units. The PCR program started at 96°C for 3 min, then 96°C for 30 s denaturation, 56°C for 50 s annealing, 72°C for 2 min extension and a final extension at 72°C for 10 min. The cycles were increased from 29 to 35. PCR product of 5μL was run on 1% agarose gel [50] to compare the existence of amplified DNA bands in SA treated and control mycelia.

### Statistical analysis

The values were represented as means of three replicates (mean ± SD). All statistical analysis was performed with SPSS Base Version 11.5 statistical software (SPSS Inc. Chicago, IL) and GraphPad Prism software 5.03 package.

## Results and discussion

### Fungal taxol production on supplement of SA

**Fig 1:**
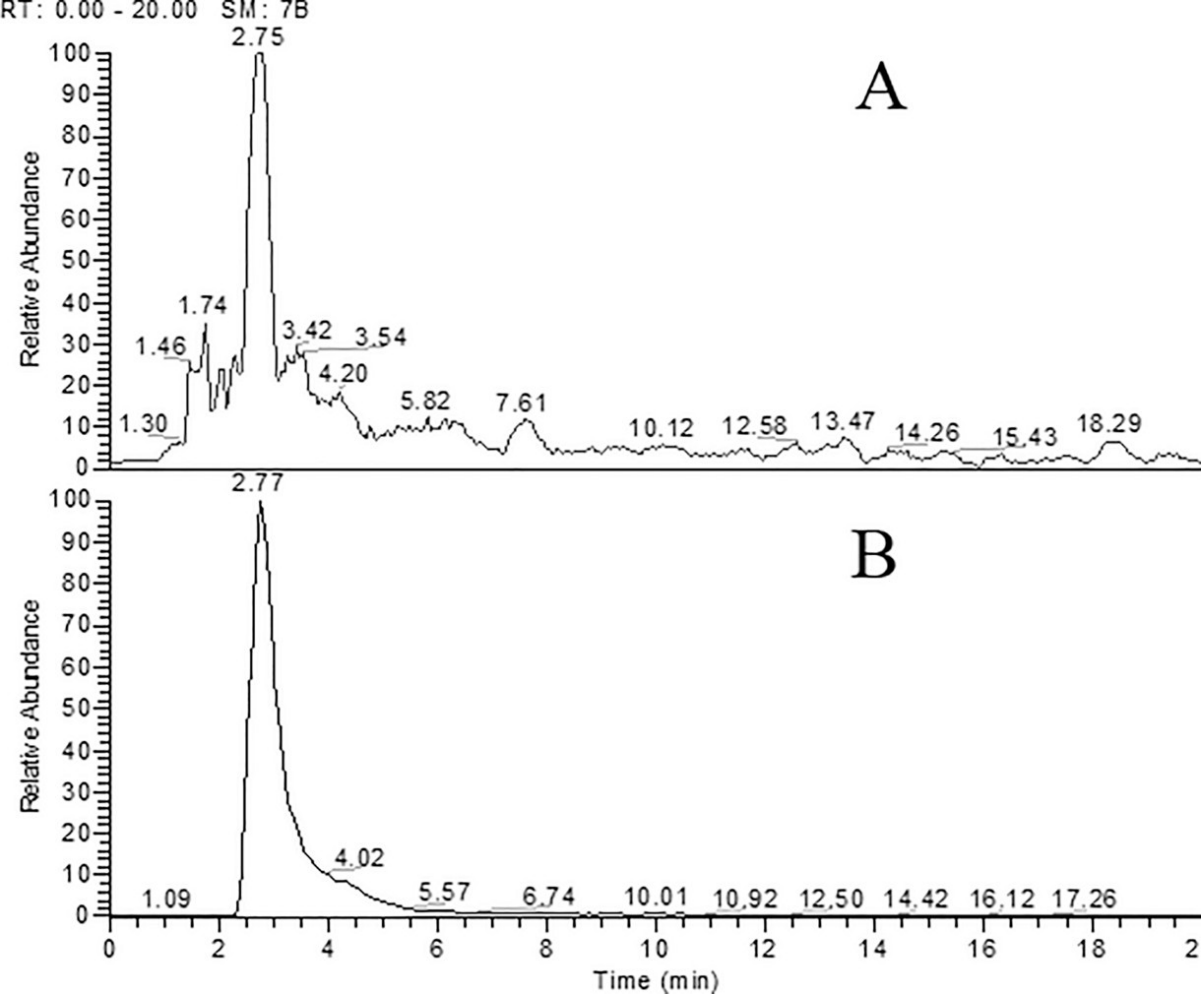
HPLC profiles of culture filtrate extract of *P*. *microspora* showing resolved peaks. Peak with R_t_ 2.75 min is a fungal taxol (A), corresponds to the standard paclitaxel R_t_ 2.77 min (B).

HPLC profiles showed the peak of taxol from the culture extract of *P*. *microspora* at a retention time of 2.75 min which is comparable with the standard paclitaxel retention time of 2.77 min (Fig 1A and 1B). Quantification of taxol was carried out based on the standard curve using standard paclitaxel.

**Fig 2:**
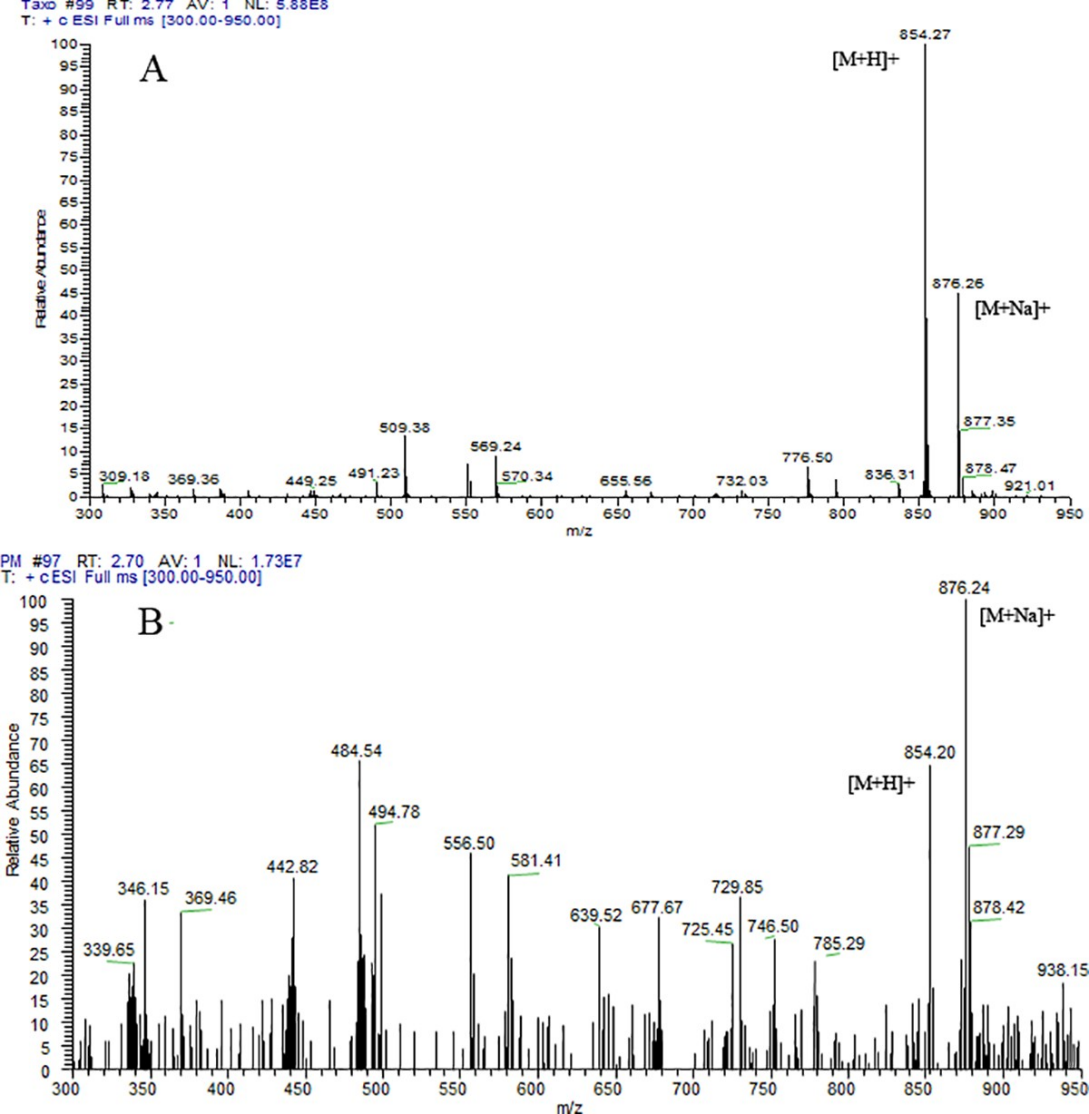
Mass spectra of taxol. (A) Standard paclitaxel. (B) Taxol from *P*. *microspora*.

The *P*. *microspora* started to produce taxol from 9^th^ day of incubation with a concentration of 74.3 μg L^-1^ of taxol (Table 1). On subsequent days, the taxol quantity gradually increased with the maximum production was 283.2 μg L^-1^ on 21^st^ day of incubation and decreased after 24 days. The presence of taxol was further confirmed by LC-ESI-MS analysis which showed fungal taxol with the molecular mass of *m/z* 854.20 [M+H]^+^ and *m/z* 876.24 [M+Na]^+^corresponding to the molecular weight of standard taxol [M+H]^+^ 854.27 and 876.26 [M+Na]^+^, respectively (Fig 2A and 2B).

**Table 1:** Fungal biomass and taxol production by *P*. *microspora* on different days and with different concentrations of SA supplementation.

Day	Biomass (g L^-1^)	Taxol (μg L^-1^)	SA (μM)	Biomass (g L^-1^)	Taxol (μg L^-1^)
3	2.34 ± 0.04^j^	0 ± 0	30	19.18 ± 0.18^k^	290.96 ± 0.82^j^
6	5.20 ± 0.10^i^	0 ± 0	60	19.69 ± 0.19^j^	304.91 ± 0.30^i^
9	8.46 ± 0.16^h^	74.28 ± 1.35^h^	75	20.23 ± 0.21^g^	317.64 ± 0.71^h^
12	10.71 ± 0.21^g^	145.96 ± 2.81^g^	120	21.22 ± 0.27^f^	330.81 ± 0.48^g^
15	13.68 ± 0.26^f^	174.52 ± 0.32^f^	150	23.24 ± 0.24^d^	377.47 ± 0.40^f^
18	18.21 ± 0.35^e^	215.37 ± 0.18^e^	240	24.28 ± 0.29^b^	413.88 ± 0.11^e^
21	22.10 ± 0.43^b^	283.18 ± 0.88^a^	300	27.30 ± 0.30^a^	625.47 ± 0.26^a^
24	23.78 ± 0.62^a^	276.83 ± 0.52^b^	600	23.26 ± 0.27^c^	607.95 ± 0.92^b^
27	21.02 ± 0.35^c^	261.67 ± 0.61^c^	1200	20.09 ± 0.19^h^	512.01 ± 0.84^c^
30	19.81 ± 0.86^d^	234.42 ± 0.19^d^	2400	19.79 ± 0.19^i^	469.65 ± 0.20^d^
-	-	-	Control	22.63 ± 0.13^e^	283.11 ± 0.78^k^

Values sharing a common letter within a column are not significant at *p*< 0.05; values are mean ± SD (n = 3)

Taxol production significantly enhanced in SA supplemented M1D medium (Table 1). The highest taxol production was observed at 300 μM of SA supplementation and the yield of taxol was 625.5 μg L^-1^, which was 45.4 fold higher than that of control 283.1 μg L^-1^. Addition of SA in the production media increased taxol production variably by the fungal strains tested [17,35,51]. Addition of 50 mg L^-1^ of SA in the production medium improved taxol yield by *Paraconiothyrium variabile* and the yield was 14.7 μg L^-1^ [34]. Another strain, *Paraconiothyrium* SSM001 displayed 2- fold increased taxol production on supplementation of SA [51]. Recently, Qiao et al. [17] reported that supplementation of CuSO_4_, sodium acetate and SA showed a significant effect on fungal taxol production by *Aspergillus aculeatus*. Furthermore, Wang et al. [52] introduced SA to plant suspension culture of *Taxus* sp. for boosting taxol production. In the present study, use of SA as an elicitor for improved production of taxol by endophytic fungus *P*. *microspora* enhanced taxol yield to 45-fold, the highest yield reported from microorganisms.

### Biomass of *P*. *microspora*

The biomass of *P*. *microspora* reached maximum on 24^th^ day of incubation (23.8 g L^-1^). The growth increased and reached up to 27.3 g L^-1^ at 300 μM and then declined (Table 1). There was a noticeable reduction in the mycelial biomass of 19.8 g L^-1^ when SA increased up to 2400 μM. Wu et al. [25] reported that growth of *Fusarium oxysporum* was notably suppressed in a liquid culture supplemented with 100 mg L^-1^ of SA. Further, Sarmadi et al. [53] reported that highest taxane production was achieved on 2% combined elicitors of SA and glucose on *Taxus baccata* callus culture. Pre-treatment of calli of *Taxus* sp. with SA also increases the biomass and enhancing the activity of antioxidant enzymes, regulating the ROS level and improving the callus tolerance to glucose [53]. However, in the present study, increased fungal biomass was recorded at low concentration of SA supplemented in the medium whereas the growth was inhibited in the addition of higher concentration of SA.

### Effect of SA on enzymes activities in relation to taxol biosynthetic pathway

**Fig 3:**
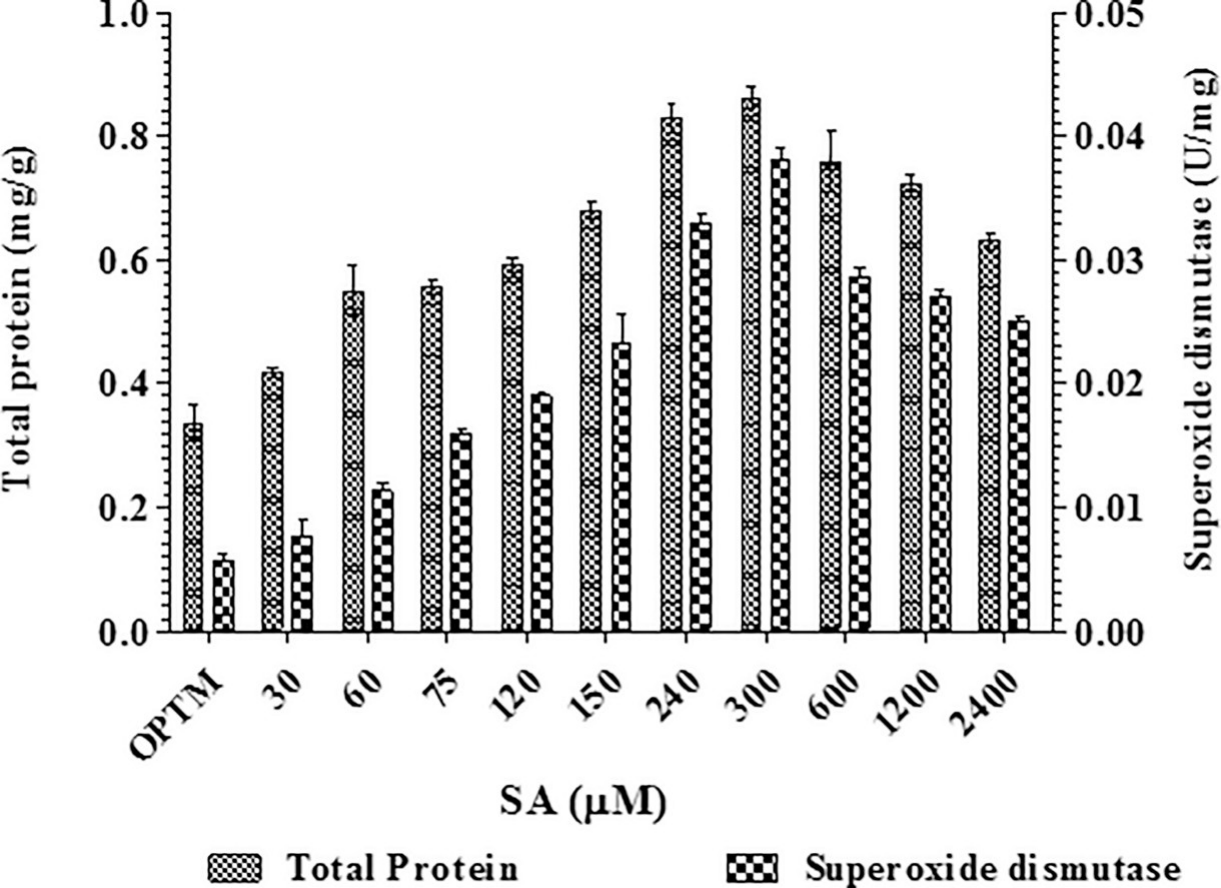
Effect of different concentrations of SA on total protein and superoxide dismutase activity by *P*. *microspora*.

The total protein content of *P*. *microspora* mycelia was increased on supplementation of SA, reached maximum 0.86 mg g^-1^ at a concentration of 300 μM SA and thereafter decreased (Fig 3). Fungi produce several antioxidant enzymes such as CAT, SOD, GSH, PX and GSH reductase to combat ROS and remove the oxygen radicals or repair the oxidative damage caused [54]. In the present study, SA generate free radicals *via* peroxidation process inducing lipid peroxidation, which involved oxidative degeneration of polyunsaturated lipids. Further, the results clearly indicate that there is no noticeable superoxide dismutase activity in *P*. *microspora* (Fig 3).

**Fig 4:**
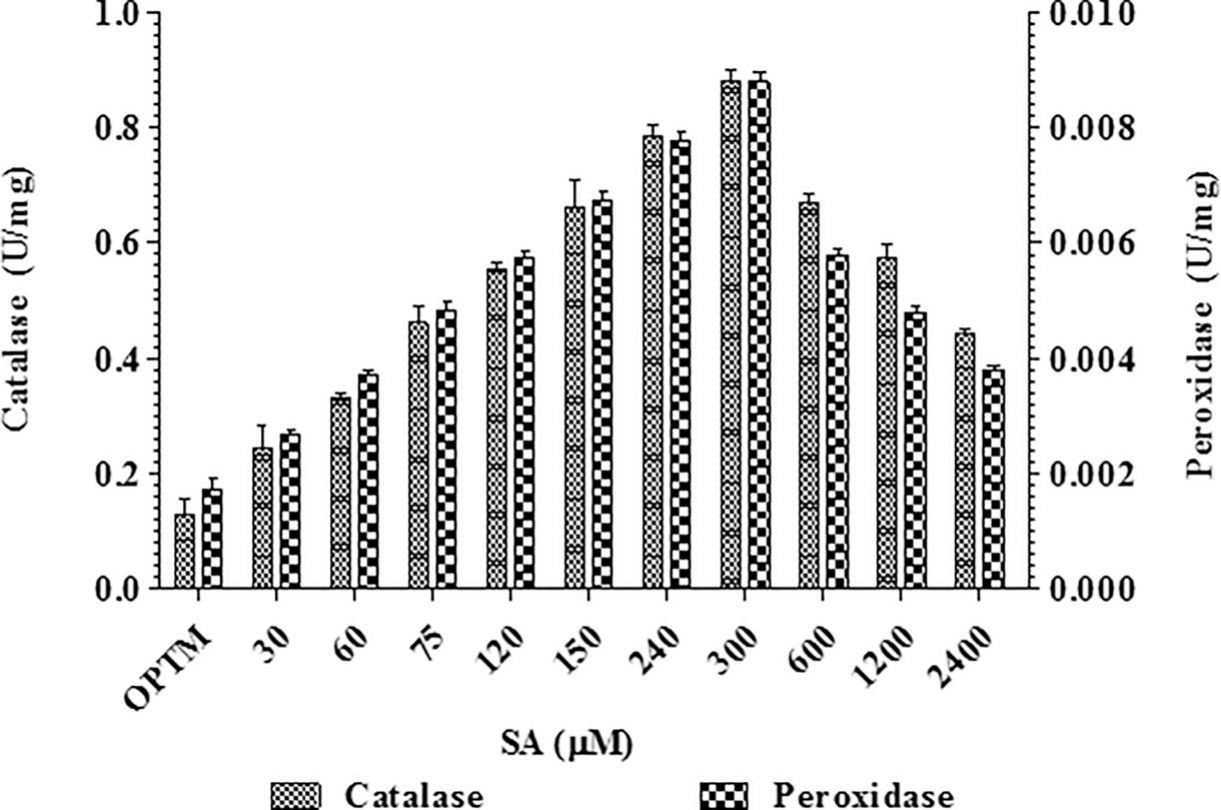
Effect of different concentrations of SA on catalase and peroxidase activity by *P*. *microspora*.

PX activity gradually increased with SA concentration from 60 to 300 μM in treated mycelia of *P*. *microspora*. The maximum activity (0.0087 U mg^-1^) was observed at 300 μM of SA in mycelia, which was less than that of untreated mycelia (0.0017 U mg^-1^) shown in the Fig 4. The peroxidase activity was 100 times less than the catalase activity. The highest catalytic activity was observed at 300 μM of SA amended mycelia which was about 0.880 U mg^-1^ of protein compared to 0.127 U mg^-1^ of protein of SA untreated sample (Fig 4). CAT is another important antioxidant enzyme that detoxifies H_2_O_2_ and converts lipid hydroperoxides to non-toxic alcohol. These systems can be induced in response to oxidative stress caused by imbalance between the production and detoxification of oxygen radicals [55].

**Fig 5:**
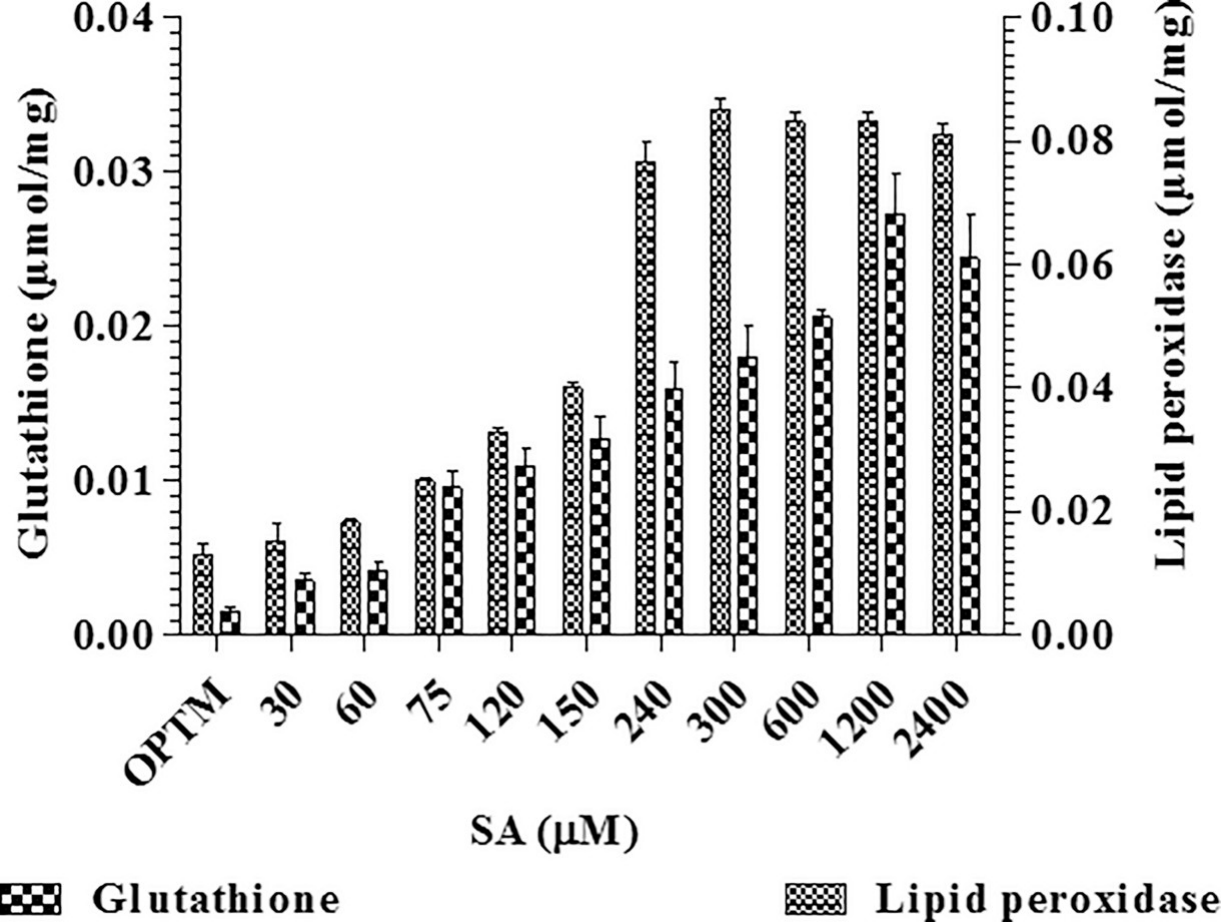
Effect of different concentrations of SA on glutathione and lipid peroxidase activity by *P*. *microspora*.

Lipid peroxidation (LPO) gradually increased with increasing SA concentrations from 60 μM to 300 μM treated mycelia with the highest LPO of 0.085 μmol mg^-1^ of recorded at 300 μM compared to control 0.012 μmol mg^-1^ of protein (Fig 5). Similarly, taxol production was increased by the addition of SA in *Taxus chinensis*, along with cell membrane-lipid peroxidation, glucose-6-phosphate dehydrogenase and peroxidase [15] and catalase [56].

**Fig 6:**
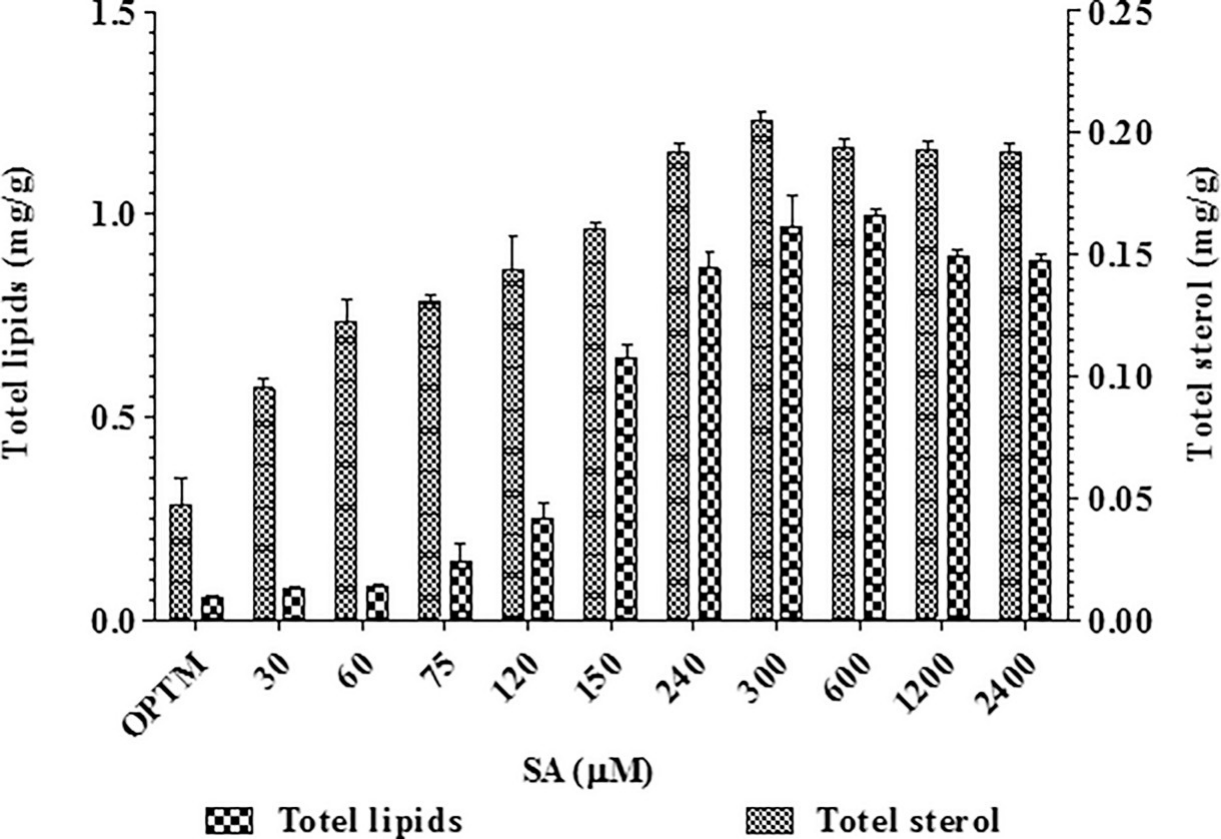
Effect of different concentrations of SA on total lipids and sterol by *P*. *microspora*.

GSH plays an important role in cellular protection from toxic effects of SA [57]. The level of GSH was 0.027 μmol mg^-1^ of protein at 1200 μM of SA treated mycelia and was quite high compared to the control 0.0015 μmol mg^-1^ of protein (Fig 5). However, lipid content gradually increased with increasing SA concentration from 60 to 300 μM, and was sustained between 600 and 2400 μM of SA treated mycelia compared to control. The highest lipid content was recorded as about 0.968 mg g^-1^ of fresh mycelia at 300 μM of SA treated mycelia compared to control 0.054 mg g^-1^ of fresh mycelia (Fig 6).

**Fig 7:**
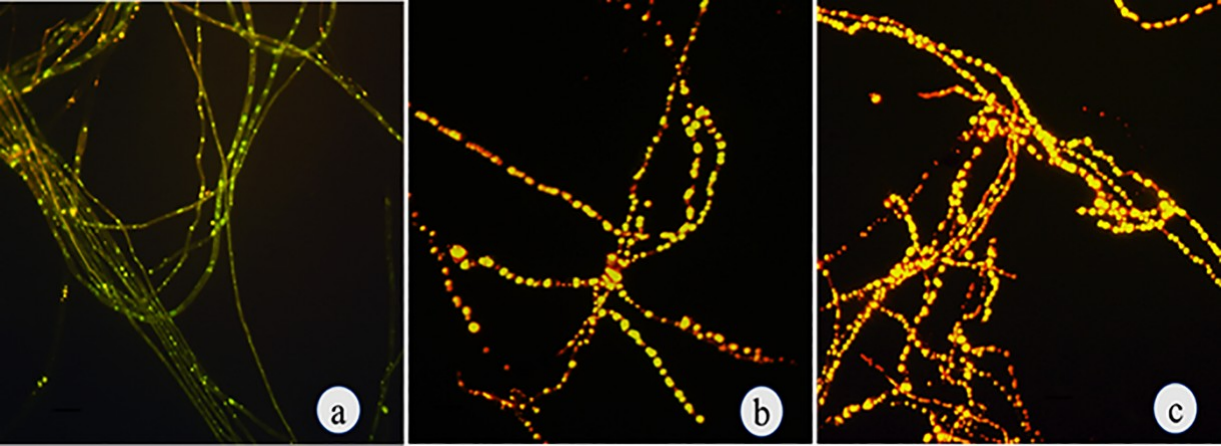
Fluorescent microscopy analysis of lipid bodies in SA treated mycelia of *P*. *microspora*. (a) Control. (b) 150 μM. (c) 300 μM.

Total fatty acids were analysed from mycelia treated with and without SA. The fatty acid 2,4-di-tert-butylphenol was found at significant amounts in control. A total of 15 fatty acids including non anoic fatty acids, hexanoic acid, 7-bromobicyclo[4,2,0]octa-1,3,5-triene, octanoic acid, octanal, undec-2-enal, nonanal and 8-methyl-1-undecene were observed in 300 μM of SA treated mycelia (S1 and S2 Tables). The sterol content in mycelia gradually increased with increasing SA concentration from 60 μM to 300 μM of SA and decreased thereafter. The highest sterol content was obtained at 300 μM SA, which was about 0.205 mg g^-1^ fresh weight of mycelia compared to control 0.046 mg g^-1^ fresh weight of mycelia (Fig 6). Furthermore, fluorescent microscopy analysis of SA treated mycelia at 150 μM and 300 μM showed higher fluorescence compared to mycelia from control, due to intracellular lipid accumulation (Fig 7). Therefore, the overall results suggested that the fungal taxol biosynthesis accompanies by lipid and sterol biosynthesis.

Isoprenoids and sterols were utilized as substrates for the accumulation of taxanes [20]. The increase in production of taxol or their intermediate baccatin III might be due to the effect of SQase on the gene expression at the initial step in the isoprenoid pathway [20]. These sterols are synthesized from mevalonic acid *via* the isoprenoid pathway [58]. Sterols and taxanes have been isolated from explants and callus cultures of *Taxus* species under elicitor MeJA treatment [59]. Therefore, it would be theoretically possible to block sterol biosynthesis, which may direct the flow of geranyl-geranyl pyrophosphate (GGPP) towards taxol biosynthesis [60].

Biochemical traits of SA treated *P*. *microspora* mycelia revealed that when lipid peroxidation increased, sterol content increased and the lipid content decreased. In addition, SOD decreased, CAT decomposes PX and high ROS lead to cellular damage. GSH plays a significant role in cellular protection from ROS at higher concentration of SA. Moreover, biomass of fungus *P*. *microspora* directly correlated with production of Taxol.

### Effect of SA on total protein content and protein expression profile

**Fig 8:**
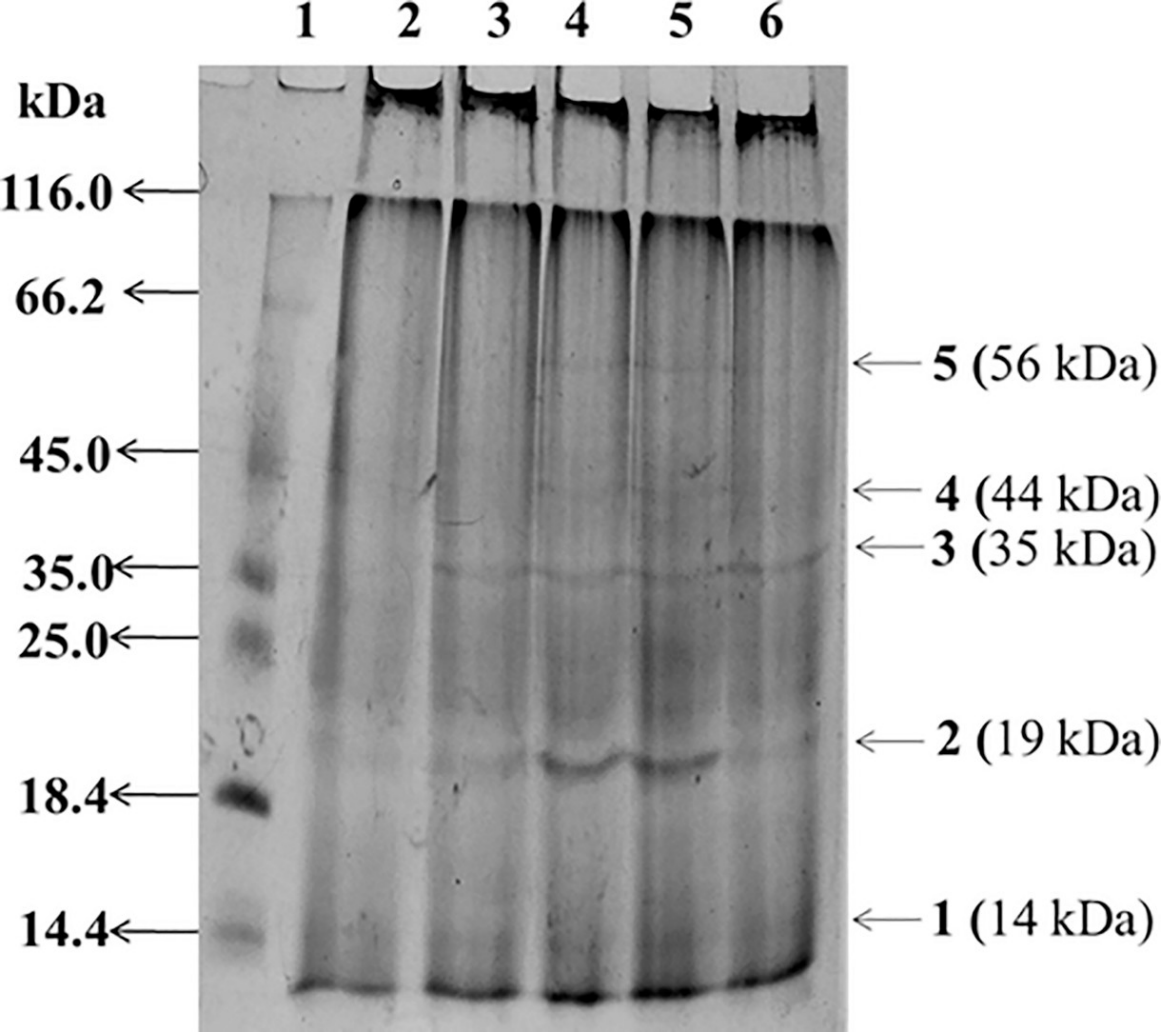
Protein profiling by SDS-PAGE showing the SA-induced changes in mycelial proteins of *P*. *microspora* analyzed. Lane 1: Marker; Lane 2: M-1D medium; Lane 3–6: 150, 250, 300 and 1200 μM, respectively of SA induced protein.

Increased concentration of SA in the M1D medium leads increased total protein content up to a certain stage. The total proteins obtained from the mycelia were loaded onto SDS-PAGE gel for primary analysis. Significant differences in the protein expression profile were observed with presumable up-regulation of certain proteins as evident from varying intensity of certain protein bands from the culture grown in M1D medium with and without addition of SA. The electrophoretic whole-cell protein patterns of SA treated and control samples showed 5 major bands per lane, within a range of molecular weight varying between 116.0 KDa and 14.4 KDa (Fig 8). Intense bands of 14, 19, 35, 44 and 56 KDa protein were observed on dendrograms from sample of mycelia with 250 and 300 μM of SA induced protein. However, the band intensity was decreased at 1200 μM of SA compared to control (Fig 8). The results showed that electrophoretic whole-cell protein patterns and their variations depend on the concentration of SA. The findings of the present study substantiated the role of SA as an elicitor and also indicated the increased total protein content in the fungal culture grown with SA. However, investigations on activity and expression of key enzymes involved in the taxol biosynthetic pathway in the presence and absence of SA will confirm the elicitation mechanism. The protein bands obtained from SA treated mycelia of *P*. *microspora* were similar to 2.5 mM SA treated protein spots previously reported in the fungus *Botrytis cinerea* [61] with clear matches of protein bands at 14, 19, 35, 44, and 56 KDa obtained in this study [61]. Further, we observed that SA induced protein expression related to the lipid metabolism and tricarboxylic acid (TCA) pathway (Fig 8). Moreover, BLAST search against *B*. *cinerea* peptide sequence confirmed the presence of biosynthetic enzymes involved in the function of virulence factor (19-kDa protein), immunity and defense (19-kDa protein), TCA pathway (34.6-kDa protein), disease/defence (34.5-kDa protein), lipid metabolism (35.2-kDa and 56.1-kDa proteins), carbohydrate metabolism (44.1 kDa), nucleic acid metabolism (45.7-kDa protein), protein metabolism and modification (55.2-kDa protein) [61].

### SA-induced expression of GGPPS by Semi-quantity RT-PCR

**Fig 9:**
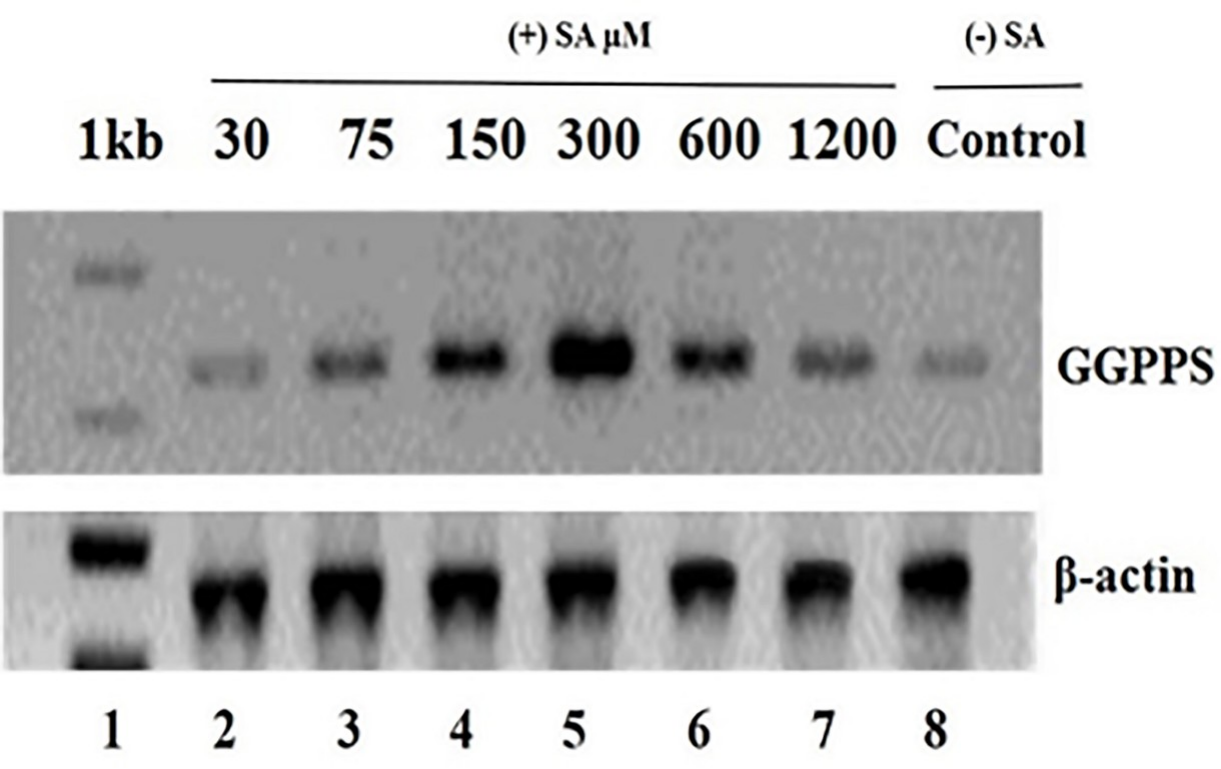
Expression profile of GGPPS after induction by SA. Total RNA was isolated from control and SA treated mycelia of *P*. *microspora* (upper panel). Actin gene was used as the control to show the normalization of quantification of RT-PCR reaction (Lower panel). The value of each concentration point is mean ± SD (n = 3). Lane 1: Marker; Lanes 2–7: 30, 75, 150, 300, 600 and 1200 μM of SA induced protein; Lane 8: control (M1D medium).

In this study, the transcript levels of GGPPS gene was studied under the condition of SA stimulation. Fig 9 showed that GGPPS gene was up-regulated after SA induction and the highest transcription level for GGPPS was observed at the optimal SA concentration. For instance, transcriptional level of GGPPS was higher at 150 μM SA, which was 2 fold higher than that of control. Besides, the maximum transcriptional level was achieved at 300 μM of SA where level was approximately 9 fold higher than that of control. Feeding terpenoid precursors isopentenyl pyrophosphate (IPP) and GGPP enhanced the taxol production 3–5 fold in an endophytic fungus *Paraconiothyrium* SSM001 [62]. Gene expression of taxol producing fungal 3-hydroxy-3-methylglutaryl-coenzyme A reductase (HMGR) gene known to be involved in taxol biosynthesis, was significantly reduced in response to light exposure [63]. Plant GGPP synthase gene positively regulate the production level of fungal taxol. Recently, an endophytic fungus *Alternaria alternata* TPF6 engineered with the modified mevalonate pathway showed increased production of taxadiene [64].

## Conclusions

The highest yield of taxol from microbial origin is reported in this study. Furthermore, this study reports for the first time on the role of SA on the biosynthetic pathway of taxol biosynthesis in fungi. The supplementation of SA increased antioxidative activities of the CAT, SOD and PX enzymes and decreased the lipid content in the culture of *P*. *microspora*. Production of peroxides in *P*. *microspora* stimulates oxidative stress that induce regulatory proteins to activate HMGR protein. The HMGR protein cascade eventually triggers GGPPS for enhanced taxol biosynthesis in *P*. *microspora*.

## Supporting information

S1 Table Total lipid profile of *P*. *microspora* from without SA amended mycelia and positive control by GC-MS analysis. (DOCX)

S2 Table Total lipid profile of *P*. *microspora* from 300 μM of SA amended mycelia by GC-MS analysis. (DOCX)
