# Supplementary material for: Salicylic acid as an effective elicitor for improved taxol production in endophytic fungus Pestalotiopsis microspora
Source: PLoS One. 2019 Feb 22;14(2):e0212736. doi: 10.1371/journal.pone.0212736 (PMC6386501; doi:10.1371/journal.pone.0212736)
Supplement: S1 Table — (DOCX) [file pone.0212736.s001.docx]

**Salicylic acid as an effective elicitor for improved taxol production in endophytic fungus *Pestalotiopsis microspora***

**Kamalraj Subban^1,3^, Ramesh Subramani^2^, Vishnu Priya Madambakkam Srinivasan^3^, Muthumary Johnpaul^3^ and Jayabaskaran Chelliah^1*^**

**1** Department of Biochemistry, Indian Institute of Science, Bangalore, 560 012, India.

**2** School of Biological and Chemical Sciences, Faculty of Science, Technology & Environment, The University of the South Pacific, Laucala Campus, Private Mail Bag, Suva, Republic of Fiji

**3** Centre for Advanced Studies in Botany, University of Madras, Guindy Campus, Chennai, Tamil Nadu, India

*Corresponding author

E-mail: cjb@iisc.ac.in (JC)

**S1 Table. Total lipid profile of *P. microspora* from without SA amended mycelia and positive control by GC-MS analysis.**

| Peak | R. time | Area | Area % | Name |
| --- | --- | --- | --- | --- |
| 1 | 13.983 | 257585 | 9.94 | N[2-(3-Bromo-Phenyl)-1-(Morpholine-4-Carbonyl)Viny |
| 2 | 13.992 | 436650 | 16.84 | Purin-2,6-dione,1,3-dimethyl-8-[2-[3.4-dimethoxyphenyl]ether |
| 3 | 14.376 | 224068 | 8.64 | 1-Methylbutyl docosanoate |
| 4 | 14.433 | 290756 | 11.22 | 2,6-Dimethylheptan-4-one |
| 5 | 17.408 | 934730 | 36.06 | 2,4-ditert-Butylphenol |
| 6 | 17.475 | 448663 | 17.31 | 4-Ethoxy-N-(1-methylacetonyl) amphetamine |
