# Supplementary material for: Salicylic acid as an effective elicitor for improved taxol production in endophytic fungus Pestalotiopsis microspora
Source: PLoS One. 2019 Feb 22;14(2):e0212736. doi: 10.1371/journal.pone.0212736 (PMC6386501; doi:10.1371/journal.pone.0212736)
Supplement: S2 Table — (DOCX) [file pone.0212736.s002.docx]

**Salicylic acid as an effective elicitor for improved taxol production in endophytic fungus *Pestalotiopsis microspora***

**Kamalraj Subban^1,3^, Ramesh Subramani^2^, Vishnu Priya Madambakkam Srinivasan^3^, Muthumary Johnpaul^3^ and Jayabaskaran Chelliah^1*^**

**1** Department of Biochemistry, Indian Institute of Science, Bangalore, 560 012, India.

**2** School of Biological and Chemical Sciences, Faculty of Science, Technology & Environment, The University of the South Pacific, Laucala Campus, Private Mail Bag, Suva, Republic of Fiji

**3** Centre for Advanced Studies in Botany, University of Madras, Guindy Campus, Chennai, Tamil Nadu, India

*Corresponding author

E-mail: cjb@iisc.ac.in (JC)


**S2 Table. Total lipid profile of *P. microspora* from 300 µM of SA amended mycelia by GC-MS analysis.**

| Peak | R. time | Area | Area % | Name |
| --- | --- | --- | --- | --- |
| 1 | 9.525 | 7625487 | 1.2 | Octanal |
| 2 | 9.608 | 10201278 | 1.61 | 7-Bromobicyclo[4,2,0]octa-1,3,5-triene |
| 3 | 9.671 | 13426502 | 2.12 | Hexanoic acid |
| 4 | 11.408 | 4087296 | 0.64 | Nonanal |
| 5 | 12.799 | 11706016 | 1.85 | Octanoic acid |
| 6 | 12.993 | 406663 | 0.06 | Dodecane |
| 7 | 13.092 | 1257643 | 0.2 | Decanal |
| 8 | 13.849 | 1003295 | 0.16 | 1.3-Di-Teri-butylbenzene |
| 9 | 13.956 | 3099354 | 0.49 | (2E)-2-Decenal |
| 10 | 14.231 | 18188594 | 2.87 | Nonanoic acid |
| 11 | 15.142 | 3487105 | 0.55 | 8-methyl-1-undecene |
| 12 | 15.199 | 1269559 | 0.2 | 1,13-Tetradeadien-3-one |
| 13 | 15.402 | 6633405 | 1.05 | Undec-2-enal |
| 14 | 15.516 | 2347312 | 0.37 | n-Decanoic acid |
| 15 | 15.863 | 1636827 | 0.26 | Tetradecane |
